# Low sphingosine-1-phosphate plasma levels are predictive for increased mortality in patients with liver cirrhosis

**DOI:** 10.1371/journal.pone.0174424

**Published:** 2017-03-23

**Authors:** Susen Becker, Benedict Kinny-Köster, Michael Bartels, Markus Scholz, Daniel Seehofer, Thomas Berg, Cornelius Engelmann, Joachim Thiery, Uta Ceglarek, Thorsten Kaiser

**Affiliations:** 1 Institute of Laboratory Medicine, Clinical Chemistry and Molecular Diagnosis, University Hospital Leipzig, Leipzig, Germany; 2 Department of Visceral, Vascular, Thoracic and Transplant Surgery, University Hospital Leipzig, Leipzig, Germany; 3 Institute for Medical Informatics, Statistics and Epidemiology, University of Leipzig, Leipzig, Germany; 4 LIFE – Leipzig Research Center for Civilization Diseases, University Hospital Leipzig, Leipzig, Germany; 5 Department of Gastroenterology and Rheumatology, University of Leipzig, Leipzig, Germany; Medical University of South Carolina, UNITED STATES

## Abstract

**Background & aim:**

The association of circulating sphingosine-1-phosphate (S1P), a bioactive lipid involved in various cellular processes, and related metabolites such as sphinganine-1-phosphate (SA1P) and sphingosine (SPH) with mortality in patients with end-stage liver disease is investigated in the presented study. S1P as a bioactive lipid mediator, is involved in several cellular processes, however, in end-stage liver disease its role is not understood.

**Methods:**

The study cohort consisted of 95 patients with end-stage liver disease and available information on one-year outcome. The median MELD (Model for end-stage liver disease) score was 12.41 (Range 6.43–39.63). The quantification of sphingolipids in citrated plasma specimen was performed after methanolic protein precipitation followed by hydrophilic interaction liquid chromatography and tandem mass spectrometric detection.

**Results:**

S1P and SA1P displayed significant correlations with the MELD score. Patients with circulating S1P levels below the lowest tertile (110.68 ng/ml) showed the poorest one-year survival rate of only 57.1%, whereas one-year survival rate in patients with S1P plasma levels above 165.67 ng/ml was 93.8%. In a multivariate cox regression analysis including platelet counts, concentrations of hemoglobin and MELD score, S1P remained a significant predictor for three-month and one-year mortality.

**Conclusions:**

Low plasma S1P concentrations are highly significantly associated with prognosis in end-stage liver disease. This association is independent of the stage of liver disease. Further studies should be performed to investigate S1P, its role in the pathophysiology of liver diseases and its potential for therapeutic interventions.

## Introduction

In 2013, liver cirrhosis led to 1.2 Mio deaths worldwide with increasing tendency [1]. Underlying pathophysiological mechanisms in progression of liver disease to cirrhosis are not yet understood.

The bioactive lysophospholipid mediator sphingosine-1-phosphate (S1P) plays major roles in various cellular processes such as proliferation, differentiation, migration and survival [2,3]. Extracellular S1P exerts its functions through activation of five specific G protein-coupled receptors (S1PR_1-5_). S1P related pathways are associated with inflammation and a number of diseases, such as cancer and cardiovascular diseases.

In circulation, S1P is mainly bound to albumin or high-density lipoprotein (HDL). Platelets [4] and erythrocytes [5,6] are major sources for blood S1P levels. In cirrhosis, platelet counts are markedly reduced and protein synthesis in the liver is eminently limited. As S1P appears to be involved in the early phase of hepatocyte proliferation [7], decreased availability of S1P might be relevant in the context of liver regeneration. Murine experiments indicate that the liver is an essential organ for maintaining normal S1P plasma levels [8]. Further, hepatocyte-derived exosomes deliver the synthetic machinery to form S1P in target hepatocytes resulting in cell proliferation and liver regeneration after ischemia/reperfusion injury or partial hepatectomy [9]. Although studies about S1P pathophysiology in liver cirrhosis have emerged recently, the influence of S1P on prognosis and finally mortality remains unclear.

To investigate the role of S1P in the context of prognosis of patients with liver diseases, the patient’s follow-up data and the short-term mortality including the results of the Model for End-Stage Liver Disease (MELD) score were analyzed.

The MELD score estimates the three-month mortality and is computed from the results of serum or plasma bilirubin, creatinine and the International Normalized Ratio (INR). This scoring system is used in many countries for prioritization of patients determined for orthotopic liver transplantation (OLT) [10]. It currently represents the best evaluated mortality score in patients with liver cirrhosis.

This study investigates for the first time circulating S1P levels and related compounds such as sphinganine-1-phosphate (SA1P) and sphingosine (SPH) in patients suffering from end-stage liver disease in relation of short term mortality.

## Material and methods

### Study population and sample collection

We collected 131 citrated plasma samples at Leipzig University hospital between December 2012 and February 2014 from patients who were evaluated for orthotopic liver transplantation (OLT). Three cases undergoing oral anticoagulation therapy with vitamin K antagonists (N = 2) or rivaroxaban (N = 1) were excluded because of their effect on the MELD score. According to the evaluation guidelines and waiting-list criteria of the Eurotransplant foundation, patients were reevaluated dependent on the MELD score after a prescribed time period. If more than one plasma samples from the same patient was available, we only included the specimen from the earliest date. Therefore, a total of 95 out of 131 citrated plasma samples from different patients were analyzed.

Patient characteristics (Table 1) and outcome data were collected with clinical data from the Leipzig University hospital’s transplant office and from electronical patient records. For patients with OLT within three months (N = 4) or one year (N = 10), patient follow-up was censored at the date of transplant surgery. For one patient who changed the transplant center within one year, follow-up was censored at transmission date. The ethics committee of the University Hospital Leipzig approved the utilization of residual citrated plasma samples for this study without an additional informed consent (ethical approval 082-10-190-42010). Researchers who meet the criteria for access to confidential data can access the data at the University Hospital Leipzig.

**Table 1:** Baseline characteristics of the analyzed study population (N = 95).

Parameter	Median (95% CI)
Gender female/male (%)	32/63 (33.7/66.3)
Age, years*	57.8 (20.4–73.7)
Deceased within one year	19 / 95 (20.0%)
Deceased within three months	09 / 95 (9.5%)
Orthotopic liver transplantation within one year	10 / 95 (10.5%)
Orthotopic liver transplantation within three months	4 / 95 (4.2%)
MELD score	12.41 (6.43–28.43)
INR, median	1.3 (1.0–2.7)
Bilirubin, μmol/L	30.3 (7.3–245.0)
Creatinine, μmol/L	78.0 (52.0–138.0)
Renal replacement therapy 2x/week	01 / 95 (1.1%)
Hemoglobin, mmol/L	7.3 (5.0–9.5)
Platelet count, exp 9/L	66.0 (26.0–103.0)
S1P, ng/mL	133.44 (58.76–252.22)
SA1P, ng/mL	33.58 (12.54–72.99)
SPH, ng/mL	6.48 (3.07–11.78)
Liver disease etiology	
- Nutritive-ethyltoxic	56 / 95 (58.9%)
- Viral hepatitis	10 / 95 (10.5%)
- Cryptogenic	16 / 95 (16.8%)
- Others**	13 / 95 (13.7%)
Comorbidity hepatocellular carcinoma	11 / 95 (11.6%)

* median (range),

** Others: Autoimmune hepatitis, acute on chronic liver failure, acute liver failure, benign liver tumors, primary biliary cirrhosis, primary sclerosing cholangitis, Budd-Chiari syndrome, Criggler-Najjar syndrome.

### Quantification of S1P, SA1P and SPH

Citrated plasma specimen, stored at -80°C until sample preparation, underwent one freeze-thaw cycle prior to analysis. Sample preparation and analyte quantification were performed according to our previously published method [11]. In brief, 15 μL of citrated plasma and 85 μL of methanol including the internal standards at concentrations of 11.8 ng/mL for C17-SPH and 588 ng/mL for C17-S1P were mixed and centrifuged. The supernatant was transferred into autosampler vials. The injection volume was 5 μL. Chromatographic separation was achieved on a SeQuant^™^ (Merck, Darmstadt, Germany) ZIC^®^-HILIC column (50 mm x 2.1 mm, 3.5 μm particle size). An API 4000^™^ LC/MS/MS system equipped with a Turbo V^™^ ion spray source operating in positive ESI mode was used for detection (Sciex, Darmstadt, Germany). S1P, SA1P and SPH were quantified by multiple reaction monitoring experiments. Chromatographic and mass spectrometric parameters can be found elsewhere [11].

Due to the 1:10 dilution with trisodium citrate solution at blood drawing (S-Monovette^®^ 3ml 9NC, Sarstedt, Nümbrecht, Germany), determined concentrations of S1P, SA1P and SPH were multiplied by 10/9.

### Laboratory data

Clinical chemistry routine parameters were available for each patient. Creatinine and bilirubin serum concentrations were determined by the application of enzymatic assay creatinine Plus Ver. 2 and Bilirubin Total DPD Gen.2 kit (both kits purchased from Roche, Mannheim, Germany), respectively according to manufacturer’s instructions. Both assays ran on the Cobas 6000 and 8000 analyzers (Roche, Mannheim, Germany). The RecombiPlasTin 2G kit (Instrumentation Laboratory, Lexington, USA) was used to determine the INR from citrated plasma using an ACL TOP 700 System (Instrumentation Laboratory, Lexington, USA).

MELD score was calculated according to the guidelines of the UNOS [12] using the following formula:

MELD score=10 * (0.957 * ln(creatinine [mg/dl])+0.378 * ln(bilirubin [mg/dl])+1.12 * ln(INR) + 0.643)

According to the guidelines, determined creatinine concentrations below 1.0 mg/dl or above 4.0 mg/dl were set on the defined minimum of 1.0 mg/dl or on the defined maximum of 4.0 mg/dl, respectively. The MELD score range is defined from 6 to 40.

### Statistics

Statistical analysis was performed using SPSS 20 (SPSS Inc., Chicago, IL, USA). We considered three-month and one-year survival. Baseline characteristics for survivors and non-survivors were provided as median and the 95% CI and compared using Mann-Whitney U Test.

For survival analysis, we applied the Kaplan-Meier Log-Rank Test and Cox Proportional Hazards Regression Model. For this purpose, the study population was divided into tertiles (T1: below the 33^rd^ percentile; T2: within the 33^rd^ and 67^th^ percentile; T3: above the 67th percentile). Tertile levels for each analyte were as follows: S1P: 33rd percentile = 110.68 ng/ml; 67th percentile = 165.67 ng/ml; SA1P: 33rd percentile = 22.67 ng/mL; 67th percentile = 50.60 ng/ml; SPH: 33rd percentile = 5.43 ng/ml; 67th percentile = 8.16 ng/ml. In addition, subgroup survival analysis was performed for cases with alcoholic liver cirrhosis (N = 56). For patients with alcoholic cirrhosis, the following tertile levels were applied: S1P: 33rd percentile = 117.44 ng/ml; 67th percentile = 157.54 ng/ml; SA1P: 33rd percentile = 32.03 ng/mL; 67th percentile = 42.31 ng/ml; SPH: 33rd percentile = 5.13 ng/ml; 67th percentile = 7.59 ng/ml. For AUROC (Area under Receiver Operating Characteristic) calculations, S1P, SA1P and SPH levels were multiplied with the factor of -1 for comparability. For correlation analysis, we used Spearman’s rank coefficients.

## Results

In our study population, 9 (9.5%) and 19 (20.0%) of 95 patients died within three-month and one-year follow-up time, respectively, without receiving an orthotopic liver transplantation (OLT). 10 of 95 patients (10.5%) received an OLT within one year, 4 of them within three months.

As shown in Table 2, concentrations of S1P and SA1P were significantly lower in patients who deceased within three-months or one-year compared to surviving patients, respectively.

**Table 2:** Baseline characteristics stratified by survival at three-months and one-year.

Parameter	Three-month outcome			One-year outcome		
	deceased	survived	p	deceased	survived	p
	Median (95%CI)	Median (95%CI)		Median (95%CI)	Median (95%CI)	
Number of patients	9	86		19	76	
Gender (m/f)	5/4	58/28	n.s.	12/7	51/25	n.s.
Age, years*	57.8 (53.7–65.3)	57.8(20.4–73.7)	n.s.	57.8 (42.7–67.2)	57.8 (20.4–73.7)	n.s.
Bilirubin, mg/dl	121.0 (36.0–245.0)	27.9 (7.3–121.0)	<0.001	51.8 (16.4–245.0)	25.8 (7.0–12.2)	<0.001
Creatinine, mg/dl	81 (44–329)	77 (54–132)	n.s.	81 (44–329)	77 (52–123)	n.s.
INR	1.9 (1.5–3.2)	1.3 (1.0–2.2)	<0.001	1.6 (1.2–3.2)	1.2 (1.0–1.7)	<0.001
MELD	23.3 (16.4–31.1)	11.5 (6.4–21.6)	<0.001	21.4 (8.5–31.1)	10.6 (6.4–19.5)	<0.001
S1P, ng/mL	82.43(38.82–139.00)	141.50 (78.22–252.22)	<0.001	91.19 (38.82–201.33)	150.14 (87.18–245.31)	<0.001
SA1P, ng/mL	18.69 (7.42–49.56)	34.99 (18.92–72.99)	0.006	23.83 (7.42–52.71)	37.32 (19.06–72.99)	0.003
SPH, ng/mL	5.38 (3.34–11.78)	6.49 (3.07–11.48)	n.s.	6.22 (3.34–11.78)	6.81 (3.07–13.23)	n.s.

*instead of 95%CI, the range is presented

**Fig 1:**
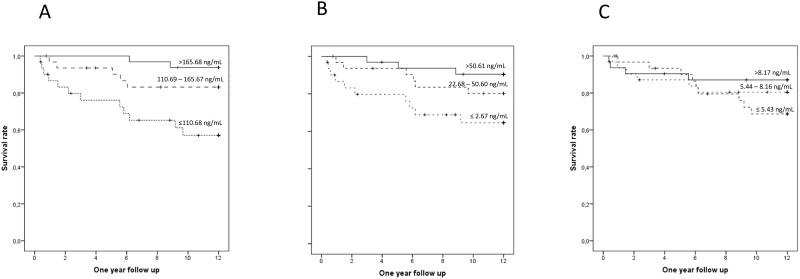
Kaplan Meier log rank tests for tertiles of S1P (A), SA1P (B) and SPH (C) for one-year survival. Censored cases are represented by a vertical line on each survival rate curve.

For further evaluation of S1P, SA1P and SPH as predictors of mortality, we performed Kaplan-Meier Log-Rank Tests for tertiles of each analyte (Fig 1).

S1P showed the highest predictive value for one-year survival. The survival rates were 93.8% for tertile 3, 83.1% for tertile 2 but only 57.1% for tertile 1. Hazard ratios (HR) of the first tertile compared with tertile 2 and 3 were 2.99 (95% CI: 1.05–8.49, p = 0.04) and 8.87 (95% CI: 1.98–39.78, p = 0.004) for one-year follow up (Table 3). Rates of survival for SA1P were 94.4% for tertile 3, 80.2% for tertile 2 and 64.5% for tertile 1. For SA1P, a significant hazard ratio of the first tertile compared with tertile 3 of 4.50 (95% CI: 1.24–16.40, p = 0.023) for one-year follow up was observed. Survival rates for SPH were 80.4%, 68.7% and 87.1% for tertile 1,2 and 3, respectively with non-significant hazard ratios between tertiles.

**Table 3:** Hazard ratios between Tertiles (T) for each analyte.

	1 year survival		
	T1T2	T1T3	T2T3
Whole study cohort (n = 95)			
S1P	2.99*	8.87**	3.02
SA1P	2.11	4.50*	2.16
SPH	0.69	1.49	2.37
Subgroup nutritive-ethyltoxic etiology (n = 56)			
S1P	3.83*	6.97*	1.69
SA1P	3.46	3.83*	1.01
SPH	0.83	1.90	2.36

*p<0.05,

**p<0.01

Cox regression for the continuous variables S1P, SA1P and SPH were only significant for S1P (three-months: HR = 0.94 per ng/mL S1P, 95% CI = 0.91–0.98, p = 0.005, one-year: HR = 0.96 per ng/mL S1P, 95% CI = 0.94–0.99, p = 0.002).

It is known that circulating S1P originates from red blood cells and platelets. In our study S1P levels correlate significantly (p<0.01, Table 4) with hemoglobin levels (spearman rank coefficient of correlation: 0.53) and platelet counts (spearman rank coefficient of correlation: 0.51), but importantly, even after including platelet counts, concentrations of hemoglobin and SA1P concentrations in the multivariate cox regression analysis S1P and SPH were significant predictors (p<0.001 and p = 0.038) of one-year mortality and S1P for three-month mortality p<0.001).

**Table 4:** Summary of spearman rank coefficients of correlation between S1P levels and liver and lipid related parameters.

	Spearman rank coefficient of correlation	p
**Liver specific parameters**		
Bilirubin (n = 95)	-0.458	< 0.001
INR (n = 95)	-0.590	< 0.001
Albumin (n = 88)	0.396	< 0.001
Cholinesterase (n = 82)	0.490	< 0.001
Aspartate aminotransferase (n = 95)	0.640	n.s.
Alanine aminotransferase (n = 95)	0.160	n.s.
y-Glutamyltransferase (n = 88)	0.186	n.s.
Creatinine (n = 95)	-0.224	0.029
**Blood related parameters**		
Platelet count	0.586	< 0.001
Hemoglobin	0.530	< 0.001
**Lipid related parameters**		
Total cholesterol (n = 77)	0.500	< 0.001
LDL-cholesterol (n = 77)	0.459	< 0.001
HDL-cholesterol (n = 77)	0.327	0.004

Values were not available retrospectively for all study subjects

We performed survival analysis for the subgroup of patients with ethyltoxic liver cirrhosis revealing comparable results. Hazard ratio for tertile 1 compared to tertile 2 or 3 were 3.83 (95% CI: 1.03–14.21, p = 0.045) for three months survival and 6.97 (95% CI: 1.50–32.49, p = 0.013) for one year survival, respectively. Due to limited sample sizes, survival analyses were not performed for other liver cirrhosis etiologies.

**Fig 2:**
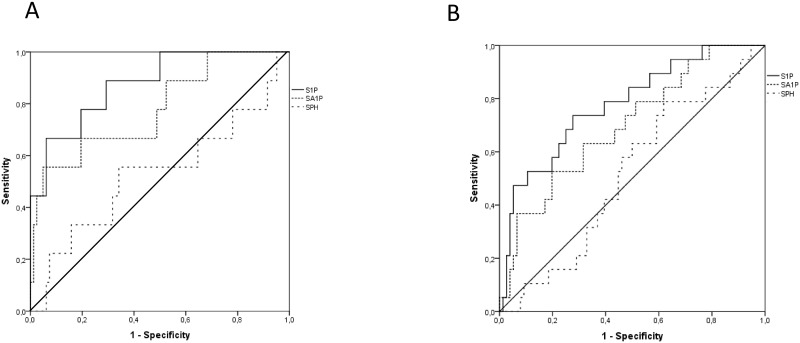
AUROCs for 3M (A) and 1Y (B) follow-up time.

Receiver operating characteristics (ROC) for three-month and one-year survival are shown in Fig 2. Compared to SA1P and SPH levels, S1P displayed the highest AUROC for three-month (0.877 vs. 0.779 and 0.528) and one-year mortality (0.883 vs. 0.782 and 0.533). The area under the curve for the MELD score was 0.949 and 0.951 for three-months and one-year mortality, respectively.

**Fig 3:**
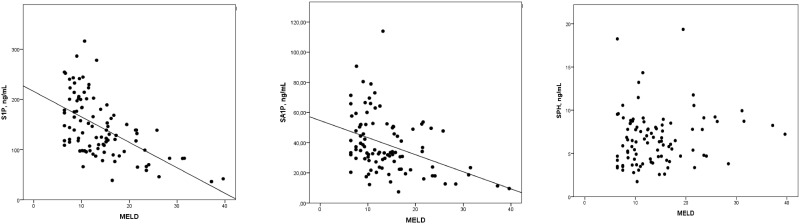
Correlation between concentrations of S1P, SA1P and SPH with the MELD score.

Levels of S1P and SA1P showed a negative correlation with the MELD score (S1P: spearman rank coefficient of correlation: -0.572, p<0.001; SA1P: spearman rank coefficient of correlation: -0.404, p<0.001, Fig 3). SPH did neither differentiate between three-month or one-year survivors and non-survivors nor was it correlated with the MELD score (spearman rank coefficient of correlation: -0.109, p = 0.109). Further correlation results between S1P levels and liver specific and lipid related parameters are summarized in Table 4.

After inclusion of the MELD score in the cox regression analysis for three-month and one-year survival, S1P remained significant (p = 0.008 and p = 0.001 vs. p = 0.003 and p = 0.004 for MELD score); indicating that S1P is a strong and independent risk predictor for mortality.

## Discussion

This is the first study linking plasma concentrations of S1P and its related compounds with mortality in a cohort of patients suffering from liver cirrhosis. There are two main findings. First, S1P is directly correlated with liver synthesis and inversely correlated with the detoxification parameter bilirubin. Secondly, low S1P plasma levels are prognostically unfavorable. Importantly, we were able to show that S1P significantly predicts mortality independently of the MELD score. In our opinion this indicates a functional role of S1P plasma levels in the progression of liver cirrhosis or in the context of cirrhosis associated complications.

In this cohort, patients with S1P plasma levels below 110.68 ng/ml had the poorest one-year survival rate of only 57.1%, whereas one-year survival rate in patients with S1P plasma levels above 165.67 ng/ml was 93.8%. Interestingly, circulating S1P concentration of the patients of this tertile were comparable with levels in a healthy population [11]. Low SA1P levels were associated with higher three-month and one-year mortality comparing survivors with non-survivors. However SA1P was not significant in the cox regression analysis.

Although we could show that sphingolipid metabolites may serve as valid mortality predictors in this study, we think that these parameters do not have the potential to serve as supplemental biomarkers in clinical routine analysis due to their sophisticated preanalytical conditions [11].

Differences in the preanalytical conditions may explain conflicting results in the literature. Two former studies investigated the involvement of circulating S1P concentrations and human liver diseases. In contrast to our data, one study detected increased serum S1P levels in patients with liver fibrosis [13]. However, due to the activation of platelets during the clotting process, serum is supposed to be an inadequate sample matrix to reflect S1P levels at the time of blood sampling [14]. Another study, including 15 patients with chronic hepatitis C infection, revealed a decrease of S1P levels with increasing progression of liver fibrosis [15], which supports our findings.

There are several possible physiological regulatory mechanisms which might explain the lower circulating concentrations of S1P in patients with a more severe degree of liver cirrhosis. First, less S1P might be released from platelets or RBCs into the blood. Previous studies showed, that platelets and RBCs are the main contributors to circulating levels of S1P [16]. Platelets are known to store high activities of sphingosine kinase which phosphorylates SPH to S1P but lack the S1P lyase preventing the degradation of S1P [17]. After platelet activation, S1P is released into the plasma. In contrast to Ikeda et al. [15], a significant direct correlation between S1P levels and platelet count as well as hemoglobin concentrations was detected in our study cohort. As shown by Cox regression analysis, S1P concentrations and even SPH predicted mortality in the multivariate analysis independently from platelet count and hemoglobin concentration.

The transportation of S1P in blood depends on carrier proteins such as apolipoprotein M (apoM) [18], being the major carrier, or albumin [19]. In healthy individuals, 60% of S1P is bound to ApoM, whereas about 30% is transported with albumin. A small fraction of S1P is bound to low density lipoproteins (LDL) and very low density lipoproteins (VLDL). However, ApoM as well as albumin are produced by the liver. Due to a decreased synthetic capacity for these proteins in the cirrhotic state, a lack of carrier proteins for S1P might occur and these ApoM-S1P and albumin-S1P complexes cannot mediate as ligands their various cellular effects. In sepsis for example, Kumaraswamy et al. identified the decreased apoM concentration as cause for the missing effect of S1P for vasculoprotection leading to vascular leakage [20].

S1P has an important impact on immune response. The S1P antagonist fingolimod is used as a potent immunosuppressive agent which is applied in several diseases such as multiple sclerosis. A lack of S1P may promote infectious complications as spontan bacterial peritonitis or sepsis in patients with severe liver disease. However, in terms of liver cirrhosis, only very few studies are available so far. First murine and in-vitro experiments showed that fingolimod inhibits cell proliferation after platelet-derived growth factor (PDGF) stimulation [21] and also suppresses migration of bone marrow-derived mesenchymal stem cells into the circulation [22] which further leads to the generation of myofibroblasts in the damaged liver [23]. The authors concluded that fingolimod might prevent progression of fibrosis of the liver. However, our data do not support this theory as lower S1P concentrations were significantly associated with higher mortality even after controlling for the MELD score. Further studies are necessary to analyze the effects on liver function in more detail.

A limitation of this study is the heterogeneous study population. We included subjects with different liver disease etiologies such as alcoholic liver disease which was the largest subgroup. Hepatocellular carcinoma was a frequent comorbidity in 11 patients. A more refined analysis of the S1P pattern according to the different etiologies of the underlying liver disease was not performed due to the limited number of patients and needs further investigation.

In conclusion, low plasma S1P concentrations are strongly associated with prognosis in end-stage liver disease, independently of MELD score. Phosphorylated sphingoid base metabolism might be a promising target for therapeutic interventions to improve the prognosis of patients of end-stage liver disease.

## Supporting information

S1 File Raw data table file. (XLSX)

## Abbreviations

AUROC: Area under Receiver Operating Characteristic
CI: confidence interval
HDL: high density lipoprotein
HR: hazard ratio
INR: international normalized ratio
LDL: low density lipoprotein
MELD: Model for End-Stage Liver Disease
OLT: orthotopic liver transplantation
S1P: sphingosine-1-phosphate
S1PR_1-5_: sphingosine-1-phosphate receptor 1–5
SA1P: sphinganine-1-phosphate
SPH: sphingosine
VLDL: very low density lipoprotein
